# Publisher’s Note

**DOI:** 10.1080/21655979.2024.2326367

**Published:** 2024-03-01

Due to a publisher error, a retraction notice was incorrectly issued on the article ‘MYCN contributes to the sensitization of acute myelogenous leukemia cells to cisplatin by targeting SRY-box transcription factor 4’, https://doi.org/10.1080/21655979.2021.1997697. We have now removed the retraction notice.

We apologise to the authors for this error.

